# Design and Optimization of Lower Limb Rehabilitation Exoskeleton with a Multiaxial Knee Joint

**DOI:** 10.3390/biomimetics8020156

**Published:** 2023-04-14

**Authors:** Jiandong Jiang, Peisong Chen, Jiyu Peng, Xin Qiao, Fengle Zhu, Jiang Zhong

**Affiliations:** 1 College of Mechanical Engineering, Zhejiang University of Technology, Hangzhou 310023, China; 2 Key Laboratory of Special Purpose Equipment and Advanced Manufacturing Technology Ministry of Education, Zhejiang University of Technology, Hangzhou 310023, China

**Keywords:** lower limb exoskeleton, gait analysis, knee movement, design, multi-objective optimization

## Abstract

To facilitate rehabilitation training for patients, we proposed the implementation of an anthropomorphic exoskeleton structure that incorporates a variable instantaneous center of rotation (ICR). This design considers the variability in knee ICR among individuals, resulting from the irregular form of the human knee joint, and leverages a double-degrees-of-freedom (2DOF) five-bar mechanism to adapt to these differences. The walking gait of the human lower limb and the corresponding knee ICR were measured and calculated using an optical 3D motion capture system. The optimal dimension parameters of the five-bar mechanism were then obtained through the optimization of human movement position inputs and rod length constraints to minimize the error in knee ICR, gait angle, and ankle trajectory between the human and the exoskeleton. Finally, we established an exoskeleton prototype to conduct relevant experimental tests. The experiment results showed that the average errors of knee ICR trajectory, hip angle, knee angle, and ankle trajectory were 5.52 × 10^−4^ m, 0.010 rad, 0.014 rad, and 1.57 × 10^−3^ m, respectively. The experimental results demonstrated that the exoskeleton’s movement trajectory was close to the human’s, reducing the human–mechanism interaction force and improving patient comfort during rehabilitation training.

## 1. Introduction

Stroke remains the third leading cause of the impairment of lower limb movement, and more than 50 million people suffer from stroke worldwide. Stroke patients must undergo high-intensity, repetitive, and frequent rehabilitation training for daily living [1,2]. In recent years, exoskeleton robots have attracted more and more attention in medical rehabilitation, assisted movement, and the military. In rehabilitation medicine, exoskeletons can provide continuous, smooth, and controlled (movement, velocity, repetition, etc.) rehabilitation training for patients with the impairment of lower limb movement. Using robotics in rehabilitation therapy can significantly increase its efficiency while reducing the labor intensity of therapists. Consequently, designing a lower extremity rehabilitation exoskeleton is imperative to assist patients in regaining lower limb movement function, thus restoring self-care abilities [3,4,5,6].

The following presents a selection of recently developed exoskeletons for people with lower extremity movement impairment. Researchers at Tsukuba University proposed a hybrid assistive limb (HAL) powered by motors and a hybrid controller [7,8,9]. The HAL is equipped with electromyography (EMG) sensors to identify the movement intentions of the wearer, as well as plantar pressure and angular sensors to capture their movement. Since 2008, the HAL has been used in various fields, such as healthcare, building construction, disaster rescue, etc. The “INDEGO” exoskeleton, developed by the Parker Hannifin Company in 2014, features a modular design that allows for convenient assembly and maintenance [10,11]. It can be used as a gait-training tool for therapy and an auxiliary mobility device to a wheelchair. The “INDEGO” exoskeleton is equipped with a mobile application that records the wearer’s movement information and can adjust the exoskeleton settings. The robotics team of the Shenzhen Advanced Technology Research Institute of the Chinese Academy of Sciences developed the “Auto LEE”, a self-balancing lower limb exoskeleton robot for high paraplegic patients [12]. “Auto LEE” consists of two robotic legs, each with five active degrees of freedom (DOFs), including hip flexion–extension, abduction–adduction, supination–pronation, knee extension–flexion, and ankle dorsiflexion–plantar flexion. With a modular structure design and multi-modal human-robot interfaces, “Auto LEE” is applicable to users of different dimensions in various conditions. It can assist patients in walking and capture the state of the exoskeleton in real-time. These rigid exoskeleton robots have anthropomorphic hip, knee, and ankle joints, and they reduce the strain on lower limbs through their support frame during rehabilitation. Additionally, flexible assist devices have proliferated in recent years, such as the Soft Exosuit from Harvard University (Massachusetts, USA) [13], HTRIUS GmbH’s BionicBack (Germany) [14], LSRO’s EXiO (Lausanne, Switzerland) [15], etc. These exoskeletons enable patients to walk naturally and provide comfortable rehabilitation exercises.

Lower limb exoskeletons designed for patients with movement impairments are typically classified into two categories based on their structural design: rigid and flexible. Although these lower limb exoskeletons can be used for various rehabilitation aids, both present particular issues. Rigid exoskeleton robots simplify knee movement as a uniaxial rotation, whereas from a bionic perspective, knee joint movement is a multiaxial rotation, presenting a J-shaped curve [16,17,18,19]. Moreover, rigid mechanical components are designed to fit tightly to the patient’s limbs, which can generate discomfort during rehabilitation exercises and may cause secondary injury to wearers, due to movement differences between the support frame and body. Flexible exoskeleton devices cannot provide stabilizing support for patients with lower extremity movement disorders, making them less optimal. To improve discomfort caused by the uniaxial knee joint, the team from Beihang University once proposed equivalenting the knee joint to two prismatic pairs and one revolute pair [20]. The overall knee joint has three active components, resulting in a complex mechanism. Furthermore, researchers from Brazil proposed a knee exoskeleton based on the crossed four-bar linkage mechanism, which boasts an ICR error of less than 3 mm [21]. This exoskeleton is equipped with a magneto-rheological clutch that decouples the motor reducer from the mechanism, allowing for high back-drivability and superior mechanical characteristics. Additionally, the instantaneous center technique has also been applied in the field of knee joint prostheses [22], usually using a four-bar mechanism to equivalent the instantaneous center movement. A four-bar mechanism optimized through parameters can well simulate knee joint movement for different individuals. However, since prostheses are customized while exoskeletons are aimed at the general public, the adaptability of four-bar mechanisms is relatively poor for exoskeletons. By comparing the mechanical structures of various rehabilitation exoskeletons, it can be concluded that current lower extremity exoskeletons still have problems, such as movement deviation and poor rehabilitation comfort for the uniaxial exoskeleton and poor adaptability for the multiaxial exoskeleton. Therefore, designing a lower limb exoskeleton more similar to human movement is essential to improve biomimetic performance.

Wearing a suitable exoskeleton can be effective for patients conducting rehabilitation exercises. However, most existing uniaxial lower limb rehabilitation exoskeletons have fixed mechanical characteristics and lack kinematic compatibility relative to the wearer’s joints, leading to inadequate bionic performance. Additionally, recent multiaxial mechanisms lack adaptability to different people. In response to these issues, a novel lower extremity rehabilitation exoskeleton is proposed in this paper, utilizing a five-bar mechanism to improve bionic performance. The knee joint of the proposed mechanism features multiaxial rotations, which are designed in a J-shaped curve to reduce motion deviation during rehabilitation therapy. Furthermore, the knee exoskeleton can be adjusted by two inputs of the five-bar mechanism to adapt to different ICR trajectories, owing to the variety of human knee contact surfaces. To ensure the mechanism aligns with the natural movement of the human knee, we considered the motion deviation of the knee ICR, the gait angle of the calf, and the movement trajectory of the ankle joint between the exoskeleton and the human movement during parameter optimization. These crucial design considerations enhance the movement precision of this mechanism for application in rehabilitation, providing significant advantages in comparison to traditional therapy methods. The main contributions are the following:

1. Analyze the physiological structure of human lower limb joints and measure the corresponding movement.
2. Propose a novel approach for designing lower limb exoskeletons and conduct a multi-objective optimization on dimension parameters.
3. Establish an exoskeleton prototype and conduct relevant experimental tests to verify the validation of the proposed approach.

## 2. Mechanical Design and Kinematic Analysis of Lower Limb Rehabilitation Exoskeleton

The exoskeleton robots should be designed based on human motion to improve their bionic performance. Thus, the physiological structures of human lower limb joints were analyzed, and the kinematic chain of the human lower extremity was established. Then, an anthropomorphic lower limb exoskeleton that included one DOF at the hip, two DOFs at the knee, and one at the ankle was proposed. Lastly, the kinematic model of the exoskeleton for optimization design was established.

### 2.1. The Physiology Analysis of Human Lower Extremity Joints

**Figure 1:**
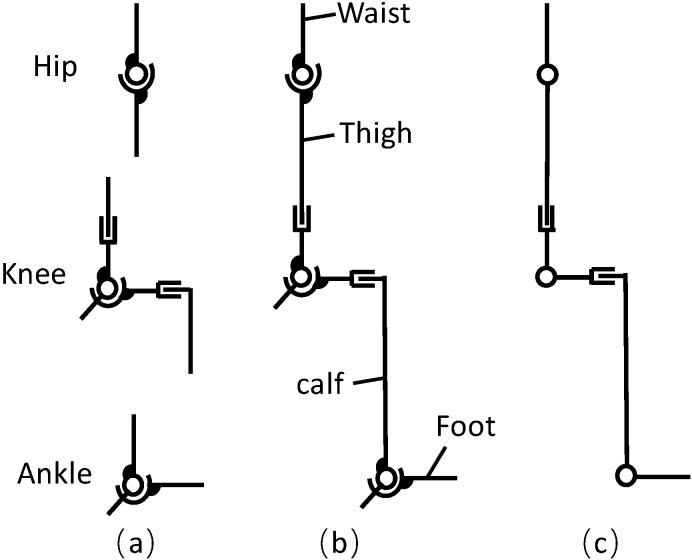
Kinematic model of a human. (**a**) Joint kinematic model; (**b**) the kinematic chain of the human lower limb; (**c**) the simplified kinematic model of the human lower limb.

The hip joint is a ball-and-socket synovial joint between the head of the femur and the acetabulum of the pelvis, and it plays an essential role in supporting the body’s weight and keeping the body in balance. The hip muscles act on three mutually perpendicular axes, all passing through the center of the femoral head, resulting in the rotation with three DOFs. Consequently, the hip joint can be modeled as a spheric joint. The knee joint is the largest joint and one of the most complex joints in the body. It is a modified hinge joint, also known as a pulley joint. The knee joint movement is classified into flexion and extension in the sagittal plane and internal and external rotation in the horizontal plane, which can only conduct when the knee joint is flexed [23]. The knee joint’s motion in the sagittal plane involves rolling and sliding, making it a multiaxial rotation joint. The knee joint’s ICR trajectory generally forms a J-shaped curve, allowing the knee to be equivalent to a joint with two revolute and prismatic pairs. The ankle includes three joints: the talocrural joint, the subtalar joint, and the inferior tibiofibular joint. Its primary function is facilitating the foot’s movement upwards, downwards, and laterally [24]. The ankle joint movements include eversion and inversion, dorsiflexion, and plantarflexion, which can be modeled as two revolute pairs. Figure 1a illustrates the kinematic model of each joint.

According to the simplified model of each joint, the kinematic chain of the human lower limb is shown in Figure 1b. The movements during walking mainly occur in the sagittal plane, so we simplified the lower extremity kinematic model as a planar kinematic model (Figure 1c). As a result, we streamlined the hip joint and ankle joint as one DOF revolute joint, and the knee joint was considered equivalent to a joint with one revolute pair and two prismatic pairs.

### 2.2. The Mechanical Design of Lower Limb Rehabilitation Exoskeleton

**Figure 2:**
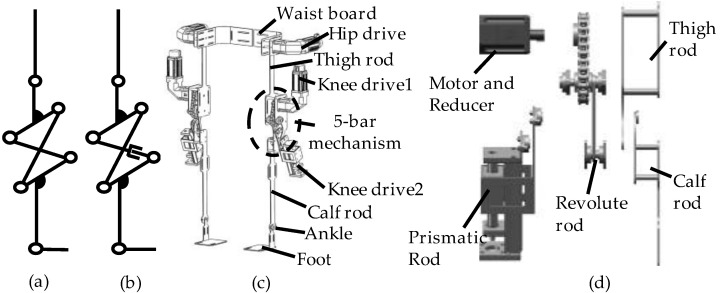
Design of the exoskeleton. (**a**) The kinematic sketch of the initial design; (**b**) the kinematic sketch of the final design; (**c**) the mechanical model of the exoskeleton; (**d**) the exploded view of the knee exoskeleton.

The four-bar mechanism has the kinematic characteristics of a variable instantaneous center, which produces a J-shaped curve trajectory, so the kinematic model can be adjusted to Figure 2a. Although it performs well in fitting the ICR of the human knee joint after optimization, its kinematic model lacks adaptability to the variations in knee ICR, which result from the irregular form of the human knee joint among individuals. This issue can be resolved by employing a 2DOF five-bar mechanism, as shown in Figure 2b. The five-bar mechanism can generate various ICR trajectories by adjusting its two inputs, thus accommodating individual differences in the knee joint structure.

The lower extremity exoskeleton design comprised two robotic legs, each consisting of a waist plate, thigh rod, knee five-bar mechanism, calf rod, foot, and three drive devices. Figure 2c shows the detailed structure of the designed lower extremity exoskeleton. We set one DOF in the hip joint and two DOFs in the knee joint. The ankle joint, on the other hand, was a passive joint. The hip exoskeleton was a serial mechanism of the motor, reducer, thigh rod, and frame. The knee exoskeleton was a five-bar mechanism, and its exploded view is shown in Figure 2d. The prismatic rod was driven by a linear actuator, and the revolute rod was driven by rotating machinery. To avoid interference between the motor and the linear actuator, a chain was used to change the position of the rotating machinery. The utilization of the five-bar mechanism allowed for the adaptation to the variability in knee ICR among individuals, due to the irregular form of the human knee joint. Additionally, the lengths of the leg rods could be adjusted to accommodate different wearers. Drive devices were controlled by a programmable logic controller (PLC), and motor parameters could be set in the personal computer (PC). Inertial measurement units (IMUs) were installed on the exoskeleton to acquire real-time movement data. The control system was positioned at the rear of the waist board. The exoskeleton was equipped with mechanical caging devices that restricted joint angles to prevent the wearer from injury. We installed the straps on the waist plates, thigh rods, calf rods, and foot soles to fix the exoskeleton on the wearer. The exoskeleton could achieve flexion and extension of the hip and knee joints, and the total weight of the mechanism, including motors, was approximately 14 kg.

### 2.3. Kinematic Analysis of Lower Limb Exoskeleton

**Figure 3:**
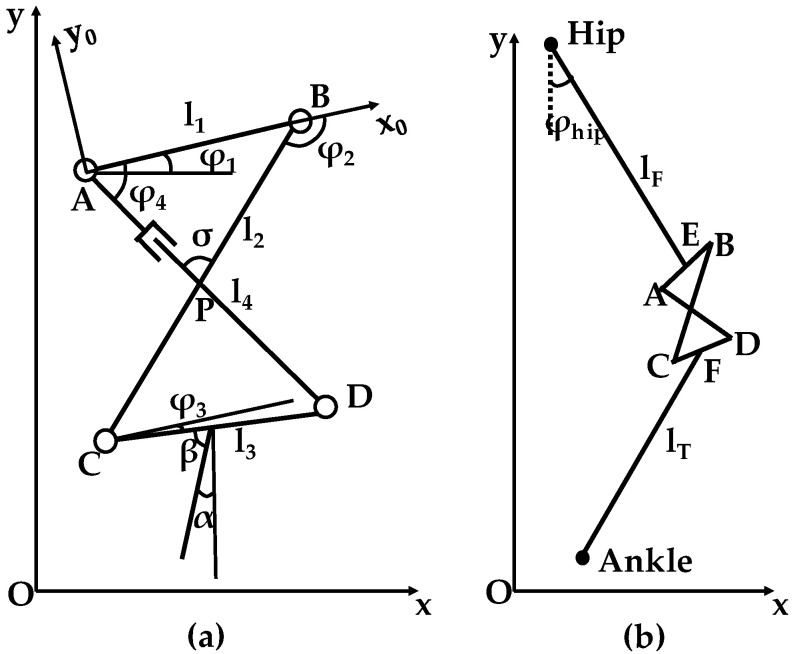
Schematic diagram of (**a**) knee joint and (**b**) lower limb exoskeleton.

The kinematic model of the knee exoskeleton was established to describe the movement position of the mechanism, as shown in Figure 3a. In this model, the thigh is fixed with rod AB, and the calf is set with rod CD. Rod BC is used for the turning part, and rod AD is used for the sliding component. The intersection point P between rod AD and rod BC is the instantaneous center of the five-bar mechanism. α represents the gait angle of the calf, and β represents the initial deviation angle between the calf and rod CD. Using this kinematic model, we can analyze and compare the deviation of ICR between the five-bar mechanism and the human knee joint during movement.

The vector equation for closed-chain motions in the A-X_0_Y_0_ coordinate system can be expressed as:

(1) $$\vec{\mathrm{AB}}+\vec{\mathrm{BC}}+\vec{\mathrm{CD}}+\vec{\mathrm{DA}}=0$$

where, $\vec{\mathrm{AB}}$, $\vec{\mathrm{BC}}$, $\vec{\mathrm{CD}}$, and $\vec{\mathrm{DA}}$ are the vectors representing the lengths and positions of rods AB, BC, CD, and AD.

Equation (1) represents the constraint that the sum of the vectors describing the lengths and positions of the rods in the closed-chain mechanism must equal zero. As a result, it can be transformed into:

(2) $$\left\{\begin{array}{l} -K_{1}\cos\varphi_{4}+K_{2}\cos\varphi_{2}+K_{3}=\cos(\varphi_{4}-\varphi_{2})\\ -K_{1}\cos\varphi_{3}-K_{4}\cos\varphi_{2}+K_{5}=\cos(\varphi_{3}-\varphi_{2}) \end{array}\right.$$

where $K_{1}=\frac{l_{1}}{l_{2}}$, $K_{2}=\frac{l_{1}}{l_{4}}$, $K_{3}=\frac{l_{1}{}^{2}+l_{2}{}^{2}+l_{4}{}^{2}-l_{3}{}^{2}}{2l_{2}l_{4}}$, $K_{4}=\frac{l_{1}}{l_{3}}$, $K_{5}=\frac{l_{4}{}^{2}-l_{1}{}^{2}-l_{2}{}^{2}-l_{3}{}^{2}}{2l_{2}l_{3}}$.

According to the double-angle formula of trigonometry, after solving Equation (2), we can obtain:

(3) $$\left\{\begin{array}{l} \varphi_{3}=2\arctan\frac{-B\pm \sqrt{B^{2}-4DE}}{2D}\\ \varphi_{4}=2\arctan\frac{-B\pm \sqrt{B^{2}-4AC}}{2A} \end{array}\right.$$

where

$A=K_{1}+K_{3}+(1+K_{2})\cos\varphi_{2}$

$B=-2\sin\varphi_{2}$

$C=-K_{1}+K_{3}+(1+K_{2})\cos\varphi_{2}$

$D=K_{1}+K_{5}+(1-K_{4})\cos\varphi_{2}$

$E=-K_{1}+K_{5}-(1+K_{4})\cos\varphi_{2}$

The movement position of the ICR of the mechanism in the O-XY coordinate system and the gait angle of the calf can be expressed as the following equations:

(4) $$\left\{\begin{array}{l} x_{P}=x_{A}+l_{AP}\cos(\varphi_{4}-\varphi_{1})\\ y_{P}=y_{A}+l_{AP}\sin(\varphi_{4}-\varphi_{1})\\ \alpha =90{}^{\circ}-\beta -(\varphi_{1}+\varphi_{3}) \end{array}\right.$$

In Equation (4), $l_{\mathrm{AP}}$ represents the distance between point A and the instant center P, which can be obtained using the sine theorem, as shown below:

(5) $$l_{AP}=\frac{l_{1}}{\sin(\left|\varphi_{2}\right|-\left|\varphi_{4}\right|)}\sin(180-\left|\varphi_{2}\right|)$$

To compare the exoskeleton movement with human motion collected by the experiment, the kinematic model of the lower extremity exoskeleton was established to describe the movement position of the exoskeleton, as shown in Figure 3b. $l_{F}$ and $l_{T}$ represent the lengths of the exoskeleton thigh and calf, respectively. Based on the kinematics model of the five-bar mechanism, the ankle trajectory of the lower extremity exoskeleton can be expressed as follows:

(6) $$\left\{\begin{array}{l} x_{\mathrm{ankle}}=x_{\mathrm{hip}}+l_{F}\cos(\varphi_{\mathrm{hip}}-90{}^{\circ})+l_{EB}\cos\varphi_{1}+l_{2}\cos(\varphi_{2}+\varphi_{1})\\ \;+l_{CF}\cos(\varphi_{3}+\varphi_{1})+l_{T}\cos(-90{}^{\circ}-\alpha )\\ y_{\mathrm{ankle}}=y_{\mathrm{hip}}+l_{F}\sin(\varphi_{\mathrm{hip}}-90{}^{\circ})+l_{EB}\sin\varphi_{1}+l_{2}\sin(\varphi_{2}+\varphi_{1})\\ \;+l_{CF}\sin(\varphi_{3}+\varphi_{1})+l_{T}\sin(-90{}^{\circ}-\alpha ) \end{array}\right.$$

where $x_{\mathrm{hip}}$ and $y_{\mathrm{hip}}$ represent the positions of the hip joints in the O-XY coordinate system.${\;\varphi }_{\mathrm{hip}}$ represents the gait angle of the hip joint.

## 3. Gait Collection and Analysis of Human Lower Limb Movement

**Figure 4:**
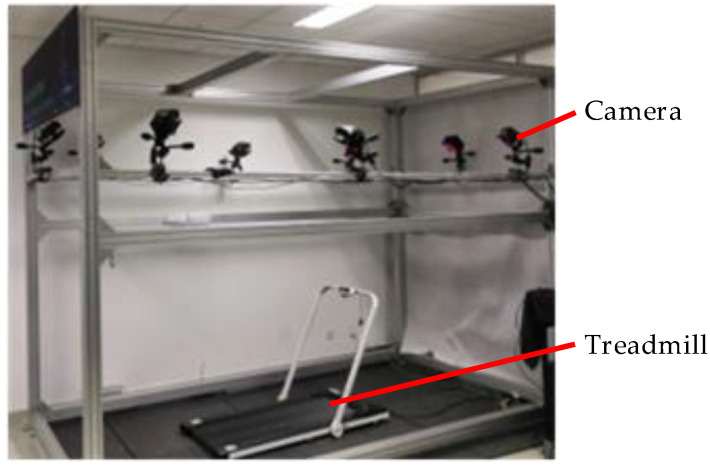
The NOKOV optical three-dimensional motion capture system.

Motion capture technology is a powerful tool for capturing the real-time movement of human bodies [25,26]. In this study, we utilized the NOKOV three-dimensional optical motion capture system (Nokov Science & Technology Co. Ltd., Beijing, China), which included eight cameras, multiple markers, and a master computer, as shown in Figure 4. The markers were coated with a reflective material that reflected the light generated by the cameras, allowing the motion capture system to collect data on moving objects’ displacement, velocity, and acceleration.

### 3.1. Collection of ICR of the Knee Joint

**Figure 5:**
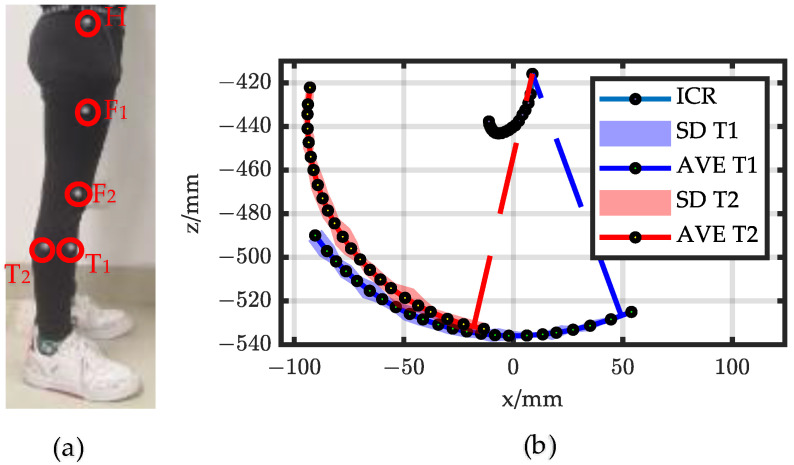
Knee ICR. (**a**) Markers layout; (**b**) movement trajectory of knee ICR.

A healthy and normal gaiter with a height of 180 cm was selected as the experimental subject. In the measurement, five markers were affixed to the subject’s lower limb, with the thigh points designated as F_1_ and F_2_, the calf points as T_1_ and T_2_, and the hip points as H, as depicted in Figure 5a. The motion of the knee joint was the rotation of the calf relative to the thigh. We defined point H as the origin of the coordinate system. Thus, the knee movement can be expressed by Equation (7).

(7) $$x_{\mathrm{Knee}}=X(T_{1},T_{2})-X(F_{1},F_{2})-X(H)$$

During the experiment, the gaiter stood at the center of the motion capture system and kept the thigh still while swinging the calf back and forth at a constant velocity. The optical motion capture system was employed to collect the coordinate positions of the markers at a sampling frequency of 100 Hz, with the data collection process lasting for 15 s and repeated five times [27]. According to the definition of the instantaneous center, the point where the relative velocity of T_1_ and T_2_ was zero was the instantaneous center of the knee joint. Notably, the ICR of a normal human knee joint varied with gait and displayed a J-shaped curve, indicating that knee motion is a composite movement of sliding and rolling, as illustrated in Figure 5b.

### 3.2. Acquisition of Human Lower Limb Walking Gait

**Figure 6:**
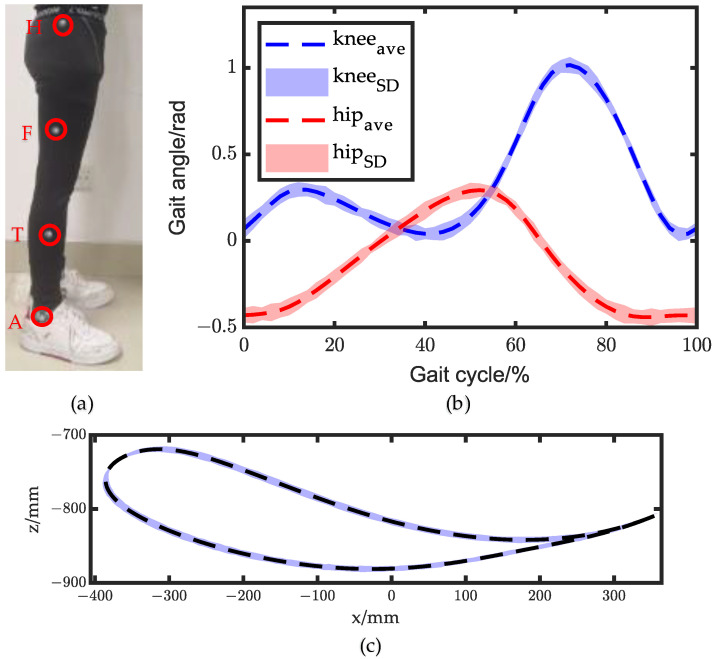
Walking gait. (**a**) Markers layout; (**b**) human walking gait angle; (**c**) ankle trajectory.

To describe the movement of the human lower extremities during walking with precision, markers were placed on the thigh and calf, and specific points were marked as the hip joint (H), ankle joint (A), thigh (HF), and calf (TA), as shown in Figure 6a. As the ankle joint was designed as a passive joint, ankle gait angle measurement was not conducted in this study. By taking the hip joint as the coordinate origin, the movement trajectory of the ankle joint was formulated as Equation (8).

(8) $$X_{\mathrm{ankle}}=X(A)-X(H)$$

As the experiment began, the subject walked on the treadmill at a velocity of 1.2 m/s, while the optical motion capture system collected the coordinate positions of the markers. The angle change in the projections of line HF and line TA on the sagittal plane expressed the walking gait angles of the thigh and calf. The human lower limb walking gait angle is shown in Figure 6b, and the trajectory of the ankle joint is shown in Figure 6c. The hip angle was measured as input for the exoskeleton hip joint, while the knee angle and ankle trajectory were obtained to assess the movement consistency between the exoskeleton and the human body.

## 4. Multi-Objective Optimization of Lower Limb Rehabilitation Exoskeleton

The more synchronized the instantaneous trajectory of the lower limb among the exoskeleton and the human is, the better the bionic performance is. From this perspective, an optimization method was utilized to obtain and optimize the dimension parameters of the lower extremity exoskeleton. Before performing the optimization algorithm, we established the objective functions for the problem [28,29,30].

During the design of the exoskeleton, various characteristics were taken into consideration, including:

1. Geometric characteristic: every part had a specific size, and there was no interference between the components.
2. Kinematical characteristic: every point on the device had a specific position, velocity, and acceleration in its movements.

### 4.1. Variables Determination

The lower extremity exoskeleton consisted of 11 basic parameters ($l_{1}$, $l_{2}$, $l_{3}$, $l_{F}$, $l_{T}$, $x_{\mathrm{hip}}$, $y_{\mathrm{hip}}$, $\varphi_{1}$, $l_{\mathrm{BE}}$, $l_{\mathrm{CF}}$, β) and two inputs ($l_{4}{}^{n}$, $\varphi_{2}{}^{n}$), as identified through kinematic analysis. ($l_{1}$, $l_{2}$, $l_{3}$, $l_{F}$, $l_{T}$) represent the rod length of the mechanism, while ($x_{\mathrm{hip}}$, $y_{\mathrm{hip}}$, $\varphi_{1}$) represent the initial position of rod AB. The positions of the thigh and calf links relative to the five-bar mechanisms are represented by ($l_{\mathrm{BE}}$, $l_{\mathrm{CF}}$, β). The variables $l_{4}{}^{n}$ and $\varphi_{2}{}^{n}$ represent the nth length and angle input. Consequently, all the variables are expressed in the following equation:

(9) $$x=\left[l_{1},l_{2},l_{3},l_{4}{}^{n},l_{F},l_{T},l_{BE},l_{CF},x_{\mathrm{hip}},y_{\mathrm{hip}},\varphi_{1},\varphi_{2}{}^{n},\beta \right]$$

### 4.2. Objective Functions and Constraints

To improve the bionic performance of the lower extremity exoskeleton, the objective functions included the following.

First, we should minimize the deviation of the knee joint between the mechanism and the human. The function of the minimization task was the summation of the Euclidean distance between the desired human ICR points and the five-bar mechanism ICR points.

(10) $$f_{1}=\sum_{i=1}^{n}{\left|P^{i}P_{0}{}^{i}\right|}^{2}=\sum_{i=1}^{n}\left[{\left(x_{P}{}^{i}-x_{P_{0}}{}^{i}\right)}^{2}+{\left(y_{P}{}^{i}-y_{P_{0}}{}^{i}\right)}^{2}\right]$$

where $P_{0}$ represents the actual knee ICR of humans.

Another objective was to align the exoskeleton knee angle with that of the human knee angle, so the second objective function was formulated as follows:

(11) $$f_{2}=\sum_{i=1}^{n}\left|\sigma_{1}{}^{i}-\sigma_{10}{}^{i}\right|$$

where ($\sigma_{1}$, $\sigma_{10}$) represent the knee angles of the lower extremity exoskeleton and the human.

The final objective function aimed to minimize the difference in ankle trajectory between the exoskeleton and the human, as shown in the following equation:

(12) $$f_{3}\equiv \sum_{i=1}^{n}{\left|a^{i}a_{0}{}^{i}\right|}^{2}=\sum_{i=1}^{n}\left[{\left(x_{\mathrm{ankle}}{}^{i}-x_{{\mathrm{ankle}}_{0}}{}^{i}\right)}^{2}+{\left(y_{\mathrm{ankle}}{}^{i}-y_{{\mathrm{ankle}}_{0}}{}^{i}\right)}^{2}\right]$$

where a and $a_{0}$ represent the ankle trajectory of the lower extremity exoskeleton and the human.

The constraints for this problem were:

(13) $$\begin{array}{l} g_{1}\equiv 2\left[\min(l_{1},l_{2},l_{3},l_{4}{}^{n})+\max(l_{1},l_{2},l_{3},l_{4}{}^{n})\right]\leq l_{1}+l_{2}+l_{3}+l_{4}{}^{n}\\ g_{21}\equiv \max(l_{1},l_{2},l_{3},l_{4}{}^{n})\leq 150\\ g_{22}\equiv \min(l_{1},l_{2},l_{3},l_{4}{}^{n})\geq 40\\ g_{3}\equiv v_{\min}<x<v_{\max} \end{array}$$

The first constraint pertained to the rod lengths required to form the four-bar mechanism. The area of the human knee joint was roughly 150 × 150 mm^2^, and to ensure the compactness of the exoskeleton, the maximum length of the links was set to 150 mm. To maintain the feasibility of the mechanism, the minimum length of the links was set to 40 mm, as indicated in the second constraint. The final constraint set the feasible range of the rest variables to make the optimization problem well-defined.

Therefore, the complete optimization problem can be formulated as the presented equation:

(14) $$\begin{array}{l} \mathrm{Objective}\;\mathrm{function}:\;F=\mu_{1}F_{1}(x)+\mu_{2}F_{2}(x)+\mu_{3}F_{3}(x)\\ \mathrm{Subject to:}\begin{array}{c} \;g_{1}(x)\\ \;g_{2}(x)\\ \;g_{3}(x) \end{array} \end{array}$$

where $\mu_{1}$, $\mu_{2}$, and $\mu_{3}$ represent the influence factors of knee ICR, gait angle, and ankle trajectory.

**Figure 7:**
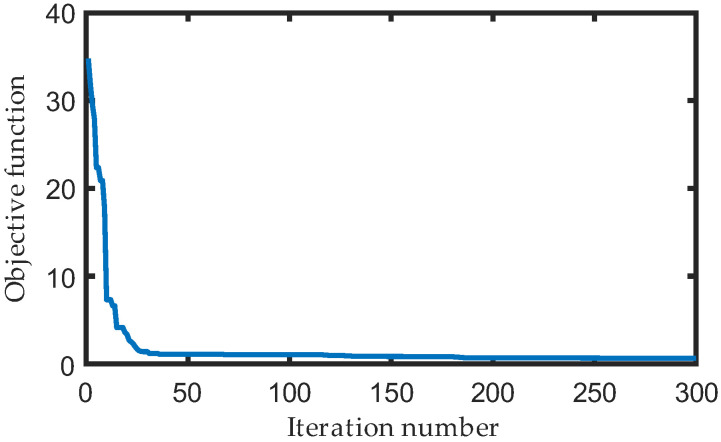
The result of the goal functions along iterations.

**Figure 8:**
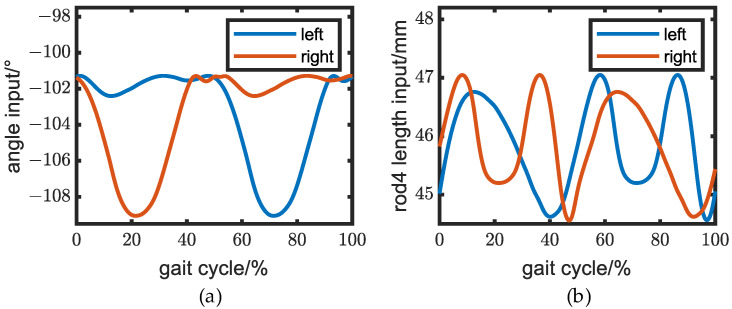
Knee inputs for the mechanisms. (**a**) Angle input and (**b**) sliding input.

Using the particle swarm optimization algorithm in MATLAB, the optimization process was carried out with the following parameters: number of individuals in the population = 100, dimension of individuals in the population = 13, maximum iteration number = 2000, and learning factor = 2. The objective function converged in approximately 180 iterations, as illustrated in Figure 7. The optimal inputs of the 2DOF five-bar mechanisms can be observed in Figure 8, while the dimension parameters of the lower extremity exoskeleton are presented in Table 1.

### 4.3. The Comparison between the Uniaxial and the Multiaxial Joints

To ensure that the gaits of both the multiaxial and the uniaxial joints were optimal, we also optimized the latter. As the ICR of the uniaxial joint was fixed, the objective equation only took into account the ankle trajectory and the gait angle.

**Figure 9:**
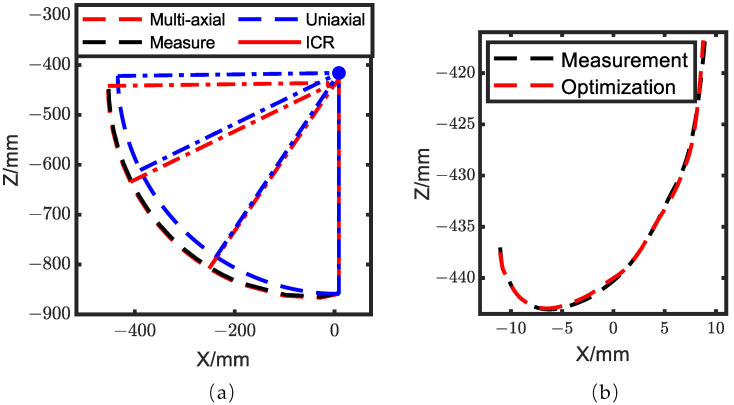
Trajectory comparison. (**a**) Ankle trajectory error of calf swing between the multiaxial and the uniaxial joints; (**b**) knee ICR movement trajectory comparison between measurement and optimization.

**Figure 10:**
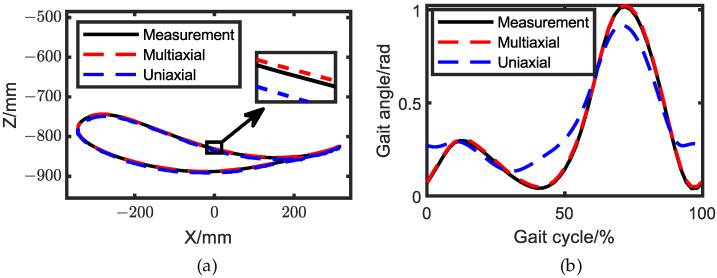
Walking trajectory comparison among measurement, the multiaxial joint, and the uniaxial joint. (**a**) Ankle trajectory; (**b**) knee gait angle.

In Figure 9a, the solid line represents the measurement of the ankle trajectory during the calf swing, while the dashed line represents the knee simulated as a uniaxial joint and a multiaxial joint. It can be observed that the multiaxial knee model was more similar to the measurement than the uniaxial knee model. When the knee angle was large, there was a considerable difference between the two joint types. Therefore, the movement of the center of rotation cannot be neglected. Figure 9b demonstrates the knee ICR trajectory of the optimization and the human measured by the motion capture system. The maximum error of the knee ICR trajectory was 6.10 × 10^−4^ m, and its average error was 3.36 × 10^−4^ m. Figure 10a describes the comparison of the ankle trajectory among the measurement, the multiaxial joint, and the uniaxial joint, while Figure 10b shows the comparison of the knee gait angle. We can observe that the walking trajectory of the multiaxial joint was more similar to the measurement than the uniaxial joint after optimization. For the multiaxial joint, the maximum error of the ankle trajectory was 7.33 × 10^−4^ m, with an average error of 3.96 × 10^−4^ m, and the maximum error of the knee gait angle was 0.012 rad, with an average error of 0.0047 rad. In comparison, for the uniaxial joint, the maximum error of the ankle trajectory was 3.33 × 10^−3^ m, with an average error of 3.12 × 10^−3^ m, and the maximum error of the knee gait angle was 0.18 rad, with an average error of 0.036 rad. The human wore the exoskeleton using straps, and these straps were equivalent to a spring-damping system, resulting in the human–mechanism coupling force being proportional to the deviation angle between the human and the exoskeleton, so the multiaxial joint had a better human–mechanism coupling force. The comparison results demonstrated that the knee ICR trajectory, ankle trajectory, and gait angle of the lower limb exoskeleton with a multiaxial knee joint were quite similar to those of humans. Therefore, using a lower limb exoskeleton with a multiaxial knee joint is beneficial for improving bionic performance.

## 5. Exoskeleton Prototype Design and Test

### 5.1. Exoskeleton Prototype Design

**Figure 11:**
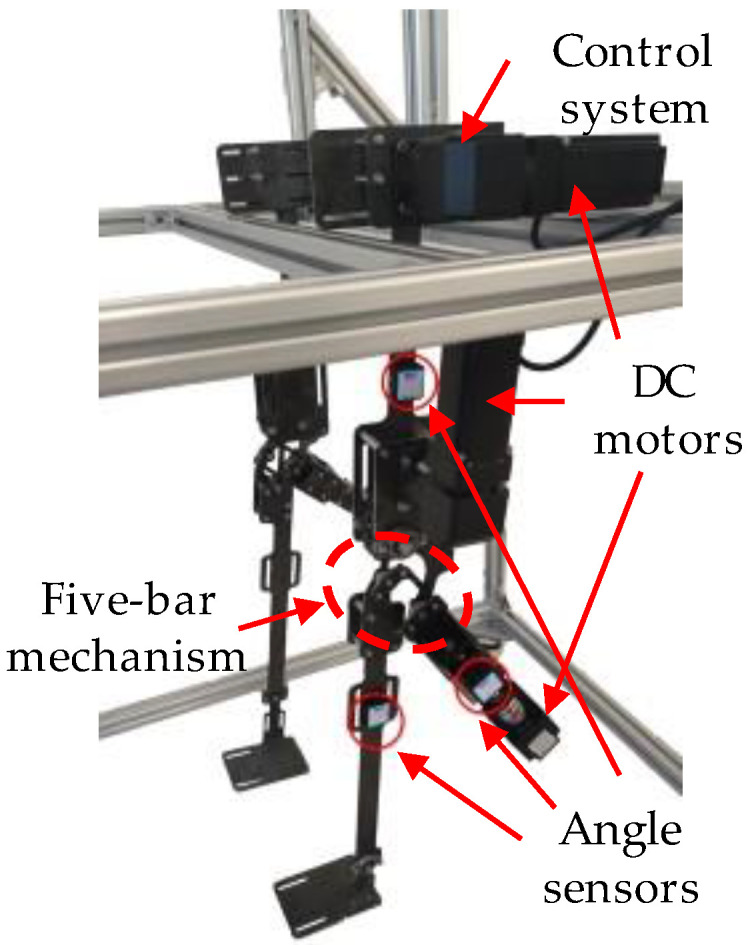
Exoskeleton prototype.

Based on the optimized structure parameters, we designed a prototype of the exoskeleton, as presented in Figure 11. The DC motors were driven through pulses generated by a PLC. To measure the motion of the exoskeleton, IMUs were installed on the mechanism, as reported in previous works [31,32]. The knee ICR and ankle trajectory were then calculated based on the measured data and dimension parameters.

### 5.2. Experimental Validation of Designed Exoskeleton

**Figure 12:**
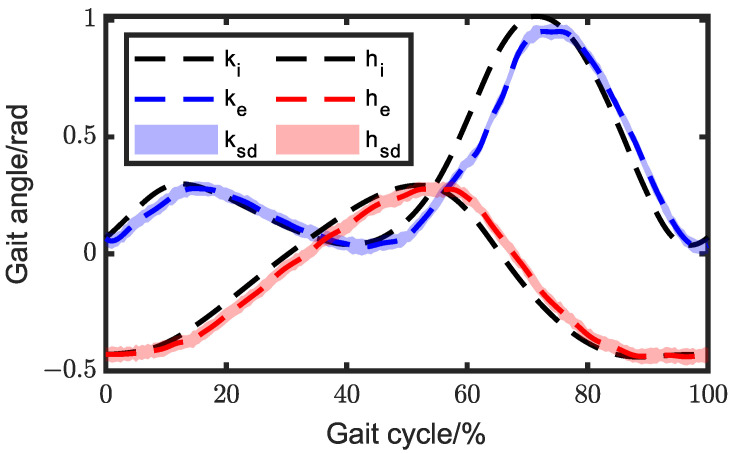
Comparison of the exoskeleton gait angle between the experimental and ideal gait angles without PID control. $k_{i}$ and $h_{i}$ represent knee and hip gait angles measured by the motion capture system; $k_{e}$ and $h_{e}$ represent the average gait angles; $k_{\mathrm{sd}}$ and $h_{\mathrm{sd}}$ represent the standard deviation.

DC motor rotational speed was set based on optimization results, and the angle sensor data was transmitted to the PC in real time. The gait angle of the exoskeleton was collected to compare with the human gait angle measured in the third part, as presented in Figure 12. The results showed that the average errors of hip angle and knee angle were 0.038 rad and 0.059 rad, respectively. Upon observation, we noted that the gait angle showed similarities to the result of the motion capture experiment, although the problem of gait incoordination persisted. This issue could be attributed to the following factors:

1. Figure 12 shows that while the DC turned around, the gait angle hardly changed for a while. This suggested the presence of clearance in the motor shaft and hole fit due to machining inaccuracies.
2. Stepper motors were used in the experiment, and they might lose pulses due to their open loop system, resulting in a slightly smaller measurement angle than the settings.

**Figure 13:**
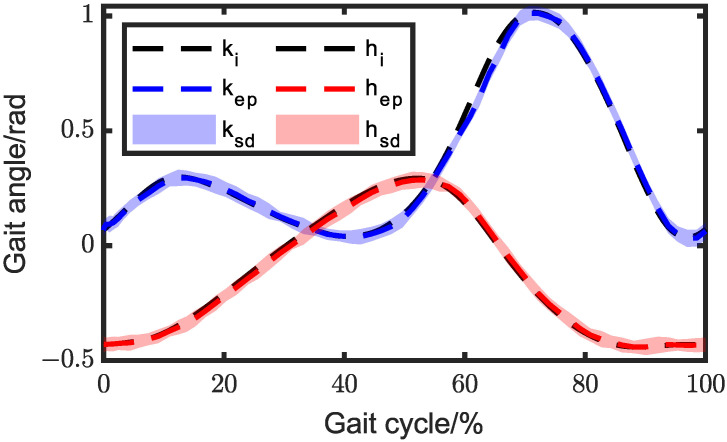
Comparison of the exoskeleton gait angle between the experimental and ideal angles with PID control. $k_{i}$ and $h_{i}$ represent knee and hip gait angles measured by the motion capture system; $k_{\mathrm{ep}}$ and $h_{\mathrm{ep}}$ represent the average gait angle; $k_{\mathrm{sd}}$ and $h_{\mathrm{sd}}$ represent the standard deviation.

**Figure 14:**
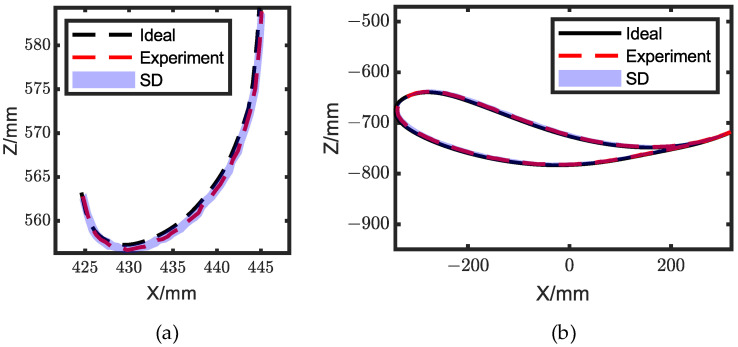
Comparison of the exoskeleton movement position between the experimental and ideal angles. (**a**) Knee ICR trajectory; (**b**) ankle trajectory.

To mitigate the angle deviation caused by the aforementioned reasons, a PID feedback control was added to the system. The gait angle of the exoskeleton with PID feedback control is depicted in Figure 13, and the average errors of hip angle and knee angle were 0.010 rad and 0.014 rad, respectively. The deviation of hip gait angle decreased by 73.68%, while the knee deviation decreased by 59.32%. The trajectories of knee ICR and ankle joint are shown in Figure 14, while the average deviations were 5.52 × 10^−4^ m and 1.57 × 10^−3^ m, respectively.

**Figure 15:**
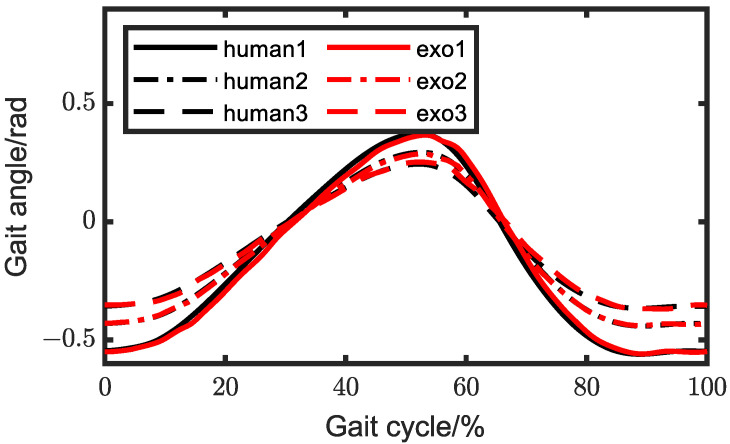
Hip-wearing experiments with different strides.

**Figure 16:**
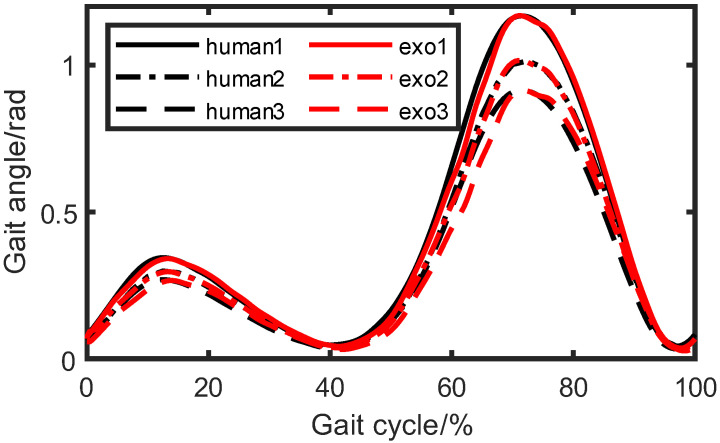
Knee-wearing experiments with different strides.

Then, we conducted experiments on the wearing of exoskeletons. Firstly, we controlled the exoskeleton walking according to the set trajectory. Considering that there might be gait angle deviations between the wearer and the exoskeleton, we also placed angle sensors on the wearer’s lower limbs to monitor the deviations in real time and compensate for the exoskeleton’s inputs. In the ICR experiment, the correspondence between gait angles and the instant center point were acquired. In multi-objective optimization, we gained the relation between the instantaneous center point and the two inputs of the exoskeleton knee joint, so the relationship between gait angles and inputs were be obtained, and the specific values of motion compensation for each input were be derived. In this experiment, we designated three strides, which corresponded to long stride, standard stride, and short stride, specifically 800, 600, and 400. The comparisons of gait angles between the exoskeleton and human are depicted in Figure 15 and Figure 16. We can observe that the gait angle of the exoskeleton was quite similar to that of a human.

To assess the exoskeleton knee ICR’s applicability to different individuals, we generated three arc curves to simulate knee ICRs for various people. According to the knee ICR measured by the motion capture system, we assumed that the ICR point when the swing angle was zero was the origin of the ICR and that the direction of the ICR origin was the negative x-axis. The following steps were taken to randomly generate an arc-shaped ICR: (1) randomly generate the coordinates of the ICR origin; (2) randomly generate the radius of the circular arc; and (3) combine the previously measured knee ICR to constrain the horizontal and vertical coordinates of the ICR endpoint, as shown in Equation (15). The parameters of the three randomly generated ICR curves are shown in Table 2.

(15) $$\left\{\begin{array}{l} x_{rs}=x_{m}\pm 2,y_{rs}=y_{m}\pm 2\\ R_{r}\in [15,40]\\ x_{rf}>-10,y_{rf}>-443 \end{array}\right.$$

**Figure 17:**
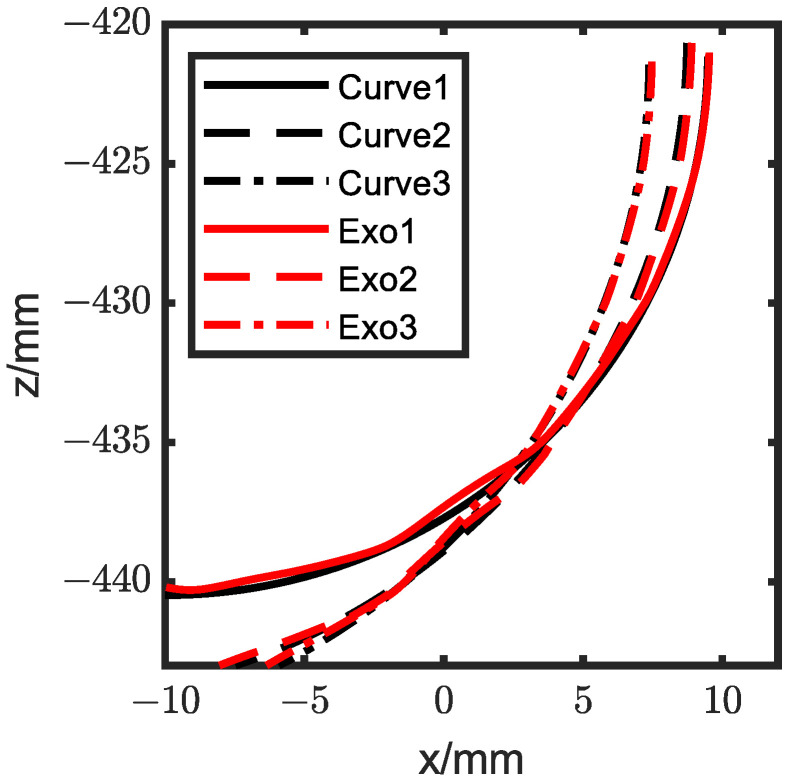
Optimization result of the adaptability experiment.

We then adjusted the inputs to recreate these knee ICRs through the five-bar mechanism, and the results are presented in Figure 17. We noted that the measured ICR closely matched the specified instantaneous center.

## 6. Conclusions

In this study, we proposed an anthropomorphic lower extremity exoskeleton based on the five-bar mechanism that was able to fit the J-shaped ICR. To improve the bionic performance of the exoskeleton, we employed a multi-objective optimization method for the dimension parameter. The exoskeleton prototype was established, and the feasibility and adaptability of the proposed exoskeleton were validated. The preliminary results indicated that the movement trajectory of the exoskeleton had the same general trend as the movement trajectory of the normal human lower limb. However, the amplitude of the buckling angle trajectories of the exoskeleton knee and hip joint models was lower than the theoretical value. To solve this problem, we increased the PID control to eliminate the deviation. Compared with the uniaxial exoskeleton, the exoskeleton with a multiaxial knee joint was beneficial for improving bionic performance. Although some DOFs of human lower limbs were simplified, the rehabilitation exoskeleton performed well in assisting wearers to walk naturally.

## Figures and Tables

**Table 1:** Parameters of the lower extremity exoskeleton.

Parameters	Value	Unit
$l_{1}$	97.34	mm
$l_{2}$	113.43	mm
$l_{3}$	59.64	mm
$l_{F}$	435.3	mm
$l_{T}$	417.23	mm
$x_{\mathrm{hip}}$	436.13	mm
$y_{\mathrm{hip}}$	1000.23	mm
$\varphi_{1}$	1.31	rad
$l_{\mathrm{BE}}$	91.18	mm
$l_{\mathrm{CF}}$	8.76	mm
β	0.26	rad
$l_{4}{}^{0}$	40.04	mm
$\varphi_{2}{}^{0}$	−1.78	rad

**Table 2:** Random knee instantaneous center trajectory parameters.

Name	Radius/mm	Arc/rad
Curve1	19.48	1.55
Curve2	23.73	1.42
Curve3	29.27	1.09

## Data Availability

Not applicable.
